# Ischemic optic neuropathy as first presentation in patient with m.3243 A > G MELAS classic mutation

**DOI:** 10.1186/s12883-023-03198-3

**Published:** 2023-04-24

**Authors:** Simone Scarcella, Laura Dell’Arti, Delia Gagliardi, Francesca Magri, Alessandra Govoni, Daniele Velardo, Claudia Mainetti, Valeria Minorini, Dario Ronchi, Daniela Piga, Giacomo Pietro Comi, Stefania Corti, Megi Meneri

**Affiliations:** 1 Neuroscience Section, Dino Ferrari Centre, Department of Pathophysiology and Transplantation (DEPT), University of Milan, Via Francesco Sforza 35, 20122 Milan, Italy; 2 Neurology Unit, Foundation IRCCS Cà Granda Ospedale Maggiore Policlinico, Milan, Italy; 3 Ophthalmological Unit, Fondazione IRCCS Cà Granda, Ospedale Maggiore Policlinico, Milan, Italy; 4 Neuromuscular and Rare Diseases Unit, Department of Neuroscience, Fondazione IRCCS Cà Granda Ospedale Maggiore Policlinico, 20122 Milan, Italy

**Keywords:** Optic neuropathy, Mitochondrial disease, MELAS, Stroke-like episodes, NAION

## Abstract

**Background:**

Mitochondrial encephalomyopathy, lactic acidosis, and stroke-like episodes (MELAS) syndrome is a systemic disorder in which multi-organ dysfunction may occur from mitochondrial metabolism failure. Maternally inherited mutations in the MT-TL1 gene are the most frequent causes for this disorder. Clinical manifestations may include stroke-like episodes, epilepsy, dementia, headache and myopathy. Among these, acute visual failure, usually in association with cortical blindness, can occur because of stroke-like episodes affecting the occipital cortex or the visual pathways. Vision loss due to optic neuropathy is otherwise considered a typical manifestation of other mitochondrial diseases such as Leber hereditary optic neuropathy (LHON).

**Case presentation:**

Here we describe a 55-year-old woman, sister of a previously described patient with MELAS harbouring the m.3243A > G (p.0, MT-TL1) mutation, with otherwise unremarkable medical history, that presented with subacute, painful visual impairment of one eye, accompanied by proximal muscular pain and headache. Over the next weeks, she developed severe and progressive vision loss limited to one eye. Ocular examination confirmed unilateral swelling of the optic nerve head; fluorescein angiography showed segmental perfusion delay in the optic disc and papillary leakage. Neuroimaging, blood and CSF examination and temporal artery biopsy ruled out neuroinflammatory disorders and giant cell arteritis (GCA). Mitochondrial sequencing analysis confirmed the m.3243A > G transition, and excluded the three most common LHON mutations, as well as the m.3376G > A LHON/MELAS overlap syndrome mutation. Based on the constellation of clinical symptoms and signs presented in our patient, including the muscular involvement, and the results of the investigations, the diagnosis of optic neuropathy as a stroke-like event affecting the optic disc was performed. L-arginine and ubidecarenone therapies were started with the aim to improve stroke-like episode symptoms and prevention. The visual defect remained stable with no further progression or outbreak of new symptoms.

**Conclusions:**

Atypical clinical presentations must be always considered in mitochondrial disorders, even in well-described phenotypes and when mutational load in peripheral tissue is low. Mitotic segregation of mitochondrial DNA (mtDNA) does not allow to know the exact degree of heteroplasmy existent within different tissue, such as retina and optic nerve. Important therapeutic implications arise from a correct diagnosis of atypical presentation of mitochondrial disorders.

**Supplementary Information:**

The online version contains supplementary material available at 10.1186/s12883-023-03198-3.

## Background

Mitochondrial encephalomyopathy with lactic acidosis and stroke-like episodes (MELAS) is one of the most common maternally inherited mitochondrial diseases [1–4].

Although childhood represents the typical age of onset, in a small percentage of cases the disease starts adulthood and is commonly associated with better prognosis and a relatively less aggressive clinical course [4]. The prevalence in the Caucasian population is reported to be around 0.24% [5].

An A to G transition in the 3243 position of mitochondrial DNA (mtDNA) is responsible for up to 80% of cases [1]. Position 3243 is placed within the MT-TL1 gene, which encodes for the mitochondrial leucine transfer RNA (tRNA) ^Leu(UUR)^. Besides that, other different pathogenic mutations have been described [1, 2, 6]. As result of the classic m.3243A > G mutation, altered mitochondrial protein translation occurs. Complex I and complex IV synthesis seems to be particularly affected, resulting in a reduced electron transport chain (ETC) function and energy deficit, especially in the most metabolically active tissues [2]. Hence, multi-organ dysfunction occurs with heterogeneous clinical manifestations, depending on the degree of mutational load harboured by the different tissues and organs, and considering respectively their energetic needs [3, 6]. Metabolically active tissues are indeed particularly affected. Neurological and muscular symptoms are the most frequent expression of the disease with epilepsy, stroke-like episodes, dementia, headache, sensorineural hearing impairment and myopathy being particularly characteristic [2, 7]. Lactic acidosis and short stature represent typical features too. Moreover, cardiac, gastrointestinal, endocrine systems can be involved as well [4].

Different plausible theories have been proposed to explain the pathogenic mechanism underlying stroke-like episodes (SLEs) in MELAS. According to a metabolic hypothesis, a local breakdown of the mitochondrial energy metabolism occurs, either in neurons, glial cells, or in cells constituting the blood brain barrier (BBB) [2, 3]. A vascular hypothesis exists as well: angiopathy seems to play an important role in the outbreak of a SLE. Regional energy deficiency can indeed stimulate mitochondrial proliferation in the smooth muscle and in the endothelial cells of small blood vessels leading to angiopathy, narrowing of the vessel diameter, thus impairing the microvasculature of several organs [2, 3]. In addition, SLE could represent a risk factor for ischemic stroke, due to a vascular impairment caused by microangiopathy [2, 3].

Increased signal on diffusion-weighted imaging represents a characteristic finding in the magnetic resonance imaging (MRI) of the acute stroke-like events, accompanied by high signal on T2-weighted and fluid-attenuated inversion recovery (FLAIR) sequences [7]. Interestingly, apparent diffusion coefficient (ADC) signal can be both increased, decreased, or mixed. This fact suggests the coexistence of cytotoxic and vasogenic edema within a SLE lesions [7].

Visual impairment represents a possible manifestation of MELAS. Retinal dystrophy, pigmentary retinopathy, ptosis, ophthalmoplegia, optic atrophy, and corneal polymegathism are well described ophthalmologic expressions of the syndrome that can cause visual defects [1, 8]. Acute visual failure in MELAS is otherwise associated with cortical blindness as a consequence of stroke-like episodes affecting the occipital cortex or other parts of the visual pathway [2, 3]. Conversely, optic neuropathy is considered a typical manifestation of other mitochondrial diseases such as Leber hereditary optic neuropathy (LHON). LHON/MELAS or LHON/MELAS/Leigh overlap syndromes are reported to exist within literature, most of all caused by uncommon mtDNA mutations [1, 8, 9]. Description of visual disturbance due to stroke-like episodes affecting the retina or the optic nerve in a carrier of the m.3243A > G MELAS canonical mutation lacks so far [10].

## Case presentation

**Fig. 1:**
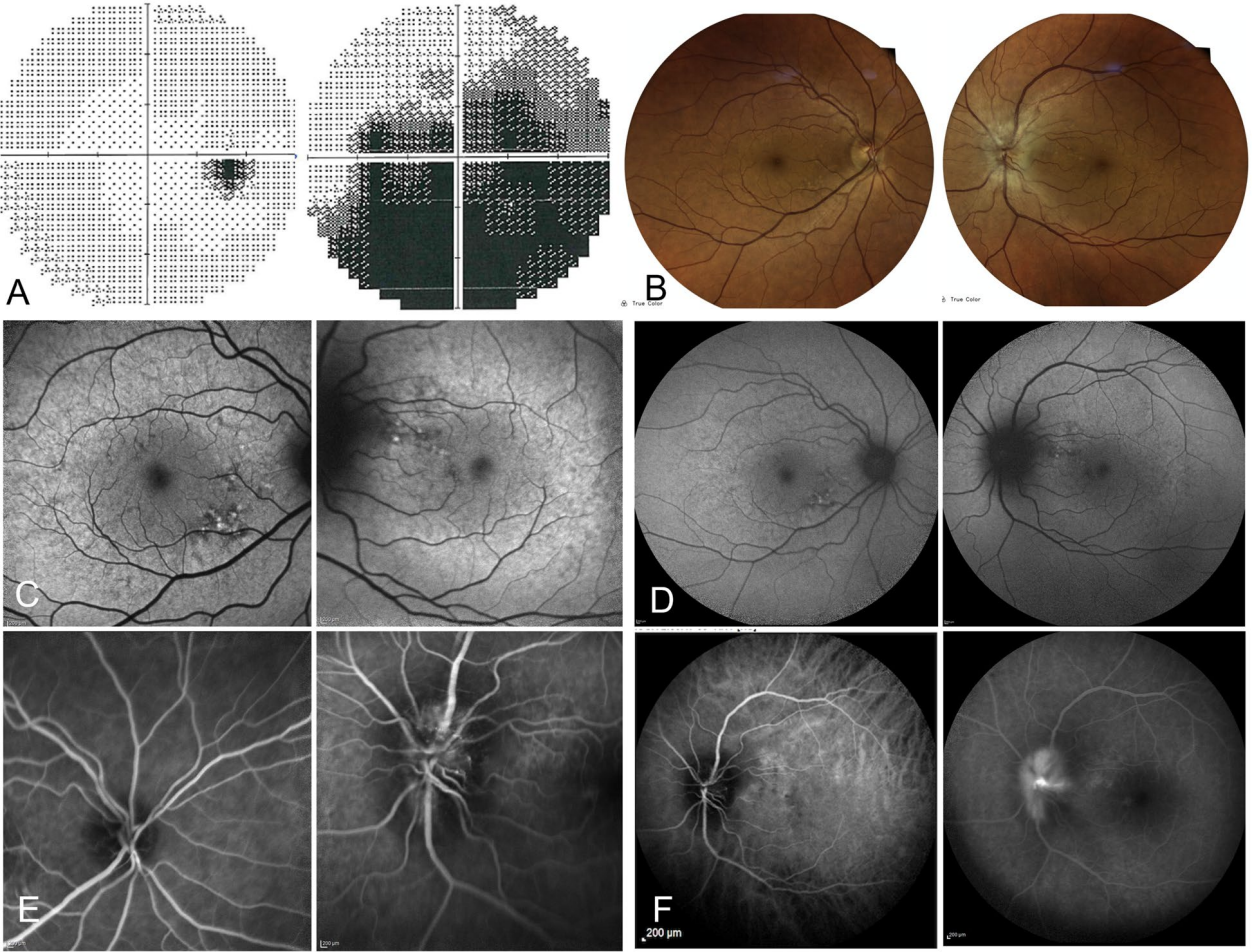
**A** Humphrey 30–2 visual field detected a normal visual field in the right eye (RE) and a dense diffuse scotoma in the left eye (LE). **B** Fundus colour picture: normal RE, LE optic disc swelling with cotton wool spots, splinter haemorrhages and venous congestion. **C**-**D** Autofluorescence images (FAF, Heidelberg Spectralis, 30 and 55 degrees lenses): bilateral mild pigmentary changes of the posterior pole, characteristic of MELAS. **E** Fluorescein angiography (FA, Heidelberg Spectralis, 30 degree lens) showed segmental perfusion delay in the optic disc, focal telangiectasia and papillary leakage, **F** Indocyanine angiography ( Heidelberg Spectralis, 55 degree lens) ruled out delayed filling of the choroid, while late phase FA ruled out other signs of ocular inflammation

**Fig. 2:**
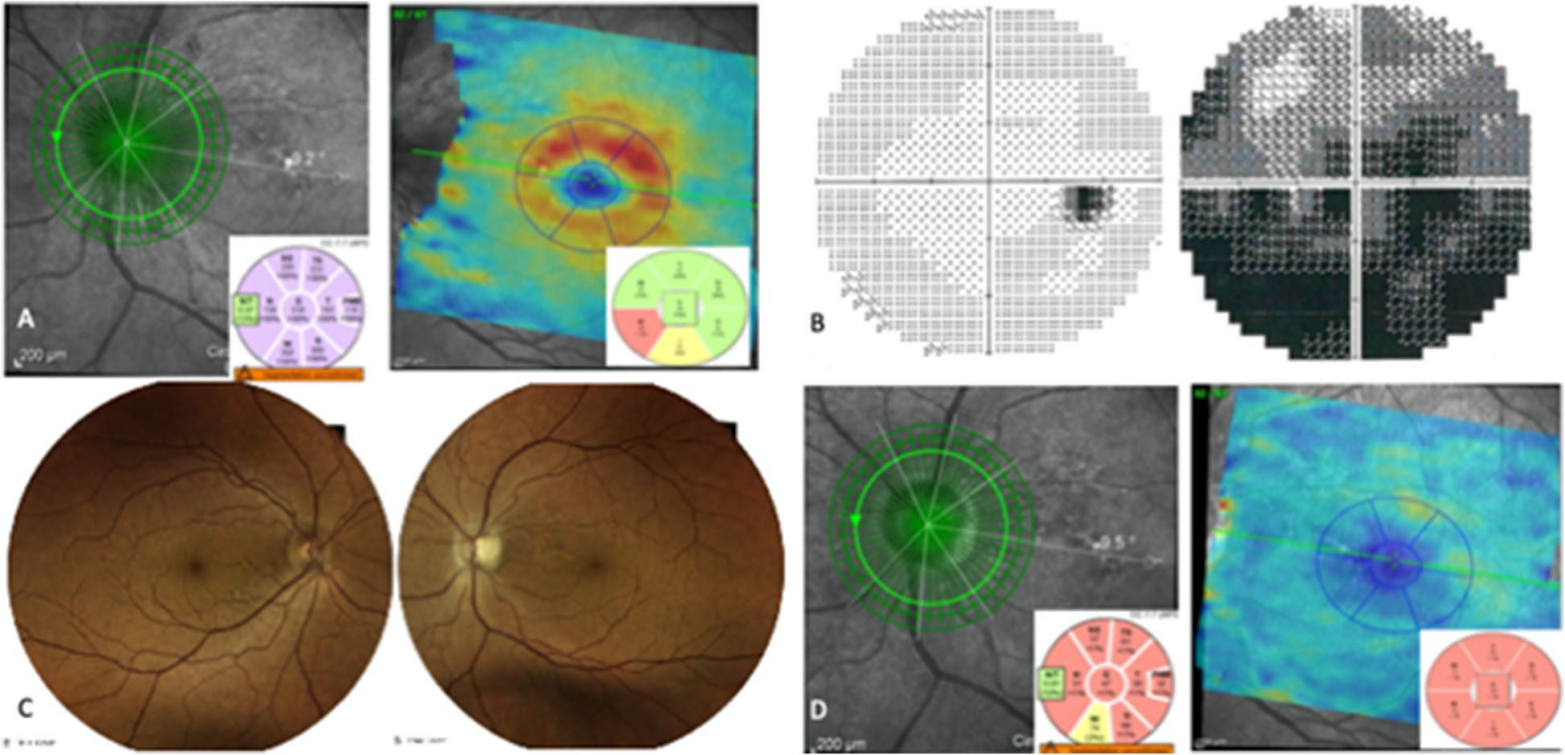
**A** Optical coherence tomography (OCT) scans detected retinal nerve fiber layer (RNFL) thickening only in the LE and an initial thinning of macular ganglion cell layer (GCL). **B** At the follow-up visit after four months, visual field defects remained almost stable, while the swelling of the optic disc resolved leaving an atrophic optic nerve head. **C** Fundus colour picture and **D** OCT after four months since the onset of symptoms

A 55-year-old woman with known medical history of arterial hypertension, hypercholesterolemia and aortic stenosis, presented with a subacute episode of visual loss started as an arcuate visual field defect in the left eye (LE) and evolved in the next days in a larger central defect. Visual symptoms were accompanied by the onset headache and diffuse muscular pain, in particular with proximal musculature involvement. Her younger brother was diagnosed a few years earlier with MELAS for relapsing episodes of intestinal pseudo-obstruction, followed by SLEs. In a short period of time he experienced a rapidly progressive cognitive decline, unfortunately leading to his death [4]. He carried a high mutational load, estimated by Real-time PCR, with an amount of 52% in muscle tissue, 28% in urinary epithelial and 18% in peripheral blood. Instead, her sister here presented resulted to be a carrier of the m.3243A > G variant, with 12% of mutation load in urinary epithelial cells and no alteration detected in peripheral blood leukocytes. She did not undergo muscular biopsy. Two days after the onset of symptoms she underwent ophthalmologic examination that revealed optic disc swelling for which prednisone 50 mg/day was started, then reduced to 25 mg/day after twelve days of therapy. One week after symptoms onset she developed left ear tinnitus, orbital pain exacerbated by ocular movements and throbbing temporal pain extended to the auricular and masseteric region worsened by chewing. Diffuse muscular pain with asthenia were still reported as well. Two weeks after the onset of symptoms the case was referred to our hospital for evaluation. She was taking oral olmesartan 40 mg daily, hydrochlorothiazide 20 mg daily, prednisone 25 mg daily. She has not had any intercurrent infections or assumption of drugs with putative mitochondrial toxicity. Therapy with ubidecarenone and L-Arginine were immediately started at admission. L-Arginine was administered intravenously for the first five days at 0.5gr/kg daily. Ubidecarenone was started and continued with a posology of 300 mg/day subdivided in three administrations. Visual acuity was 20/20 in the right eye (RE) and 20/20 in the LE. Visual field exam confirmed a dense diffuse scotoma in the affected eye (Fig. 1A). Ophthalmologic evaluation confirmed left eye optic neuropathy consisting in unilateral swelling of the optic nerve head, with cotton wool spots, splinter haemorrhages and venous congestion. The right optic disc was normal and showed a physiological excavation. Mild pigmentary changes of the posterior pole, characteristic for MELAS, were detected bilaterally (Fig. 1B). Autofluorescence images confirmed this finding (Fig. 1C,D). Fluorescein angiography (FA) showed segmental perfusion delay in the optic disc, focal telangiectasia and papillary leakage, while indocyanine angiography ruled out delayed filling of the choroid. Late phase FA ruled out other signs of ocular inflammation such as vasculitis (Fig. 1E,F). OCT scans detected retinal nerve fiber layer (RNFL) thickening only in the LE and an initial thinning of macular ganglion cell layer (GCL) (Fig. 2A). Visual evoked potential showed a reduction in amplitude with prolonged P100 in the left eye. Brain MRI did not show optic nerve inflammation or any other pathological feature, while spectroscopy revealed abnormal lactate peak in the CSF. To rule out atypical presentation of multiple sclerosis (MS), neuromyelitis optica (NMO) and neuromyelitis optica spectrum disorders (NMOSD), spinal cord MRI was also performed nineteen days after the onset of symptoms and did not show demyelinating lesions at all. Short tau inversion recovery (STIR) and diffuse weighted imaging (DWI) sequences were performed too without any findings supporting inflammatory or SLE-related lesions. Serum myelin oligodendrocyte glyco (MOG) and aquaporin auto-antibodies were found to be negative. Erythrocyte Sedimentation Rate (ESR) and C Reactive Protein (CRP) were assessed for the first time after ten days of steroids therapy and were both negative. Fludeoxyglucose positron emission tomography (FDG-PET) and temporal artery biopsy were performed without any findings that might support large-vessel vasculitis of inflammatory processes. Other possible risk factors for non-arteritic anterior ischemic optic neuropathy (NAION) such as anaemia, diabetes, nocturnal dip, sleep apnoea were investigated and excluded. Serum lactate was 1.7 mmol/L. CSF analysis was unremarkable. CSF infection was excluded and oligoclonal bands were absent. mtDNA sequencing analysis was performed and ruled out the possible presence of mitochondrial mutations known to be associated with MELAS/LHON overlap syndrome. Mitochondrial sequencing analysis confirmed the m.3243A > G transition, and excluded the three most common LHON mutations, as well as the m.3376G > A, described in literature for being associated with LHON/MELAS overlap syndrome.

**Fig. 3:**
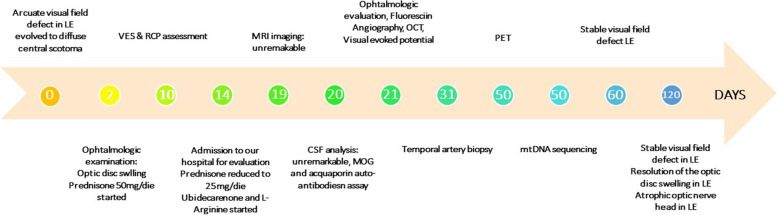
Timeline of the case report

At the follow up visit after four months, visual field defects remained stable (Fig. 2B), while the swelling of the optic disc resolved leaving an atrophic optic nerve head (Fig. 2C,D; Fig. 3).

## Discussion and conclusions

Here we present a unique phenotype of MELAS related optic neuropathy in a patient with a common m.3243A > G mutation. The visual defect and the ophthalmologic alteration compatible with ischemic neuropathy may represent the consequence of energy deficiency in the optic nerve due to a MELAS vascular related SLE. mtDNA sequencing was important to confirm MELAS diagnosis and to rule out possible mutations which account for MELAS/LHON overlap syndrome. However, some aspects regarding the onset of the visual defect were atypical for a LHON: severe unilateral optic nerve head swelling, segmental perfusion delay and leakage of the optic disc on FA were more consistent with an ischemic optic neuropathy, in addition orbital pain exacerbated by ocular movements combined with throbbing temporal pain worsened by chewing were suggestive of an arteritic aetiology. The diffuse muscular pain that she reported was a typical manifestation that could either be associated to the myopathy existing within MELAS, either to inflammatory phenomena affecting muscles during GCA. PET and temporal artery biopsy were indeed fundamental to exclude a large vessel disease vasculitis. ESR and CRP were unreliable since they were first assessed after ten days of steroids therapy. Excluding such type of vasculitis, it was possible to start prednisone titration in a patient harbouring a syndrome known to be associated with possible endocrinological dysfunction (such as diabetes) which could also be aggravated by steroids.

Although visual evoked potential showing a reduction in amplitude with prolonged P100 in the left eye represents a typical finding of demyelinating lesion affecting the optic nerve, brain MRI did not show any altered signal of the optic nerve, which would otherwise be present in the context of MS, NMO and NMOSD presentation.

SLEs are commonly accompanied by elevated lactate levels in serum and CSF analysis, along with increased lactate at MR-spectroscopy in the central nervous system (CNS) regions affected by the SLE event [2, 3, 7]. In this case, only MR spectroscopy revealed abnormal lactate peak in the CSF, not confirmed then by its physical–chemical analysis or by its serum levels assessment. Moreover, it must be considered that vasoconstriction and angiopathy might have played a more important role in the outbreak of this SLE, compared to the contribution of the disrupted mitochondrial energy production. Hypoperfusion and ischemia of the anterior optic nerve might better explain the clinical picture here described. (NAION) is manifested as isolated, sudden, nevertheless painless, monocular vision loss with oedema of the optic disc [11]. Subacute progressive worsening of vision over a period of a few days or a few weeks is a common outcome of NAION [11]. Diagnosis of NAION relies mainly on clinical features with the demonstration of vision loss with a relative afferent pupillary defect and oedema of the optic disc. Vision loss severity is highly variable and can include both visual-field defects with a preserved visual acuity but also profound vision losses. Disc pallor often replaces the oedema that typically resolves over a period of 6 to 11 weeks. Imaging of the optic nerve is one of the most important exams within the diagnostic work-up of ischemic optic neuropathy. Although the exact pathogenesis of NAION remains unproven, it appears to be a multifactorial disease. It is presumed to be due to a transient disruption in the circulation of the optic nerve head leading to hypoperfusion and ischemia. The exact cause of this transient disturbance remains unclear, but several hypotheses have been proposed including generalised hypoperfusion, nocturnal hypotension, local autoregulation failure, vasospasm, venous occlusion, and thrombosis. Systemic hypertension has been documented in up to 50% of patients [12]*.* In our patient, who presented risk factors for NAION, the perfusion of the optic disc could have been partially compromised by hypertension and worsened because of SLE secondary to a microangiopathy or transient local energetic breakdown in mitochondrial function. MELAS-like retinal anomalies detected on fundoscopy and fundus autofluorescence (FAF) imaging, further support this theory [13, 14]. Moreover, OCT reduction of GCL is an already described finding within MELAS retinal assessment [15]. Based on the constellation of clinical symptoms in our patient and the results of the investigations performed, we diagnosed an ischemic optic neuropathy in a middle-aged woman harbouring MELAS classic mutation, possibly secondary to a stroke-like event affecting the optic nerve. The pathogenesis of this SLE could be in our opinion better explained by the angiopathy existent within MELAS, thus needed to be separated from the pathogenesis of a classic NAION. In our opinion, the simultaneous onset of diffuse muscular pain, typical for myopathic involvement within mitochondrial disorders, further supports this hypothesis. L-arginine and ubidecarenone therapies were therefore started to improve stroke-like episode symptoms and prevention of new ones, as well as avoiding the involvement of the other eye. After the initiation of therapy we did not assist to further progression of symptoms or to the outbreak of new possible ones.

This is the second case of a possible SLE described in a carrier of mutation m.3243A > G with swollen optic disc; previously, a case with bilateral transient optic disc oedema was described [10]. However, differently from our case, the recovery of the visual acuity was complete. Considering that SLE may be at least partially reversible, the authors speculated that a similar phenomenon had occurred in their patient [10].

In conclusion, atypical clinical presentations must be always considered in mitochondrial disorders, even in well-described phenotypes and even if mutational load in peripheral tissue (that are easily accessible for analysis) is low. Indeed, the stochastic mitotic segregation of mtDNA molecules does not allow to foresee the exact degree of heteroplasmy existent within different tissues, such as retina and optic nerve. As a consequence, important therapeutic implications arise from a correct diagnosis of atypical presentations of mitochondrial disorders.

In conclusion, mitochondrial gene mutations may present not only with different and less frequently atypical phenotypes, but they may also be considered as an adjunctive risk factor for ischemic events. In this case, we think that better knowledge of the genetic background could help not only in a correct diagnosis, but also for a tempestive and tailored therapy.

### Supplementary Information

**Additional file 1.** Supplementary materials. Family history and pedigree.

## Data Availability

The original contributions presented in the study are included in the article/supplementary material, further inquiries can be directed to the corresponding authors.

## Abbreviations

ADC: Apparent diffusion coefficient
BBB: Blood barrier brain
CNS: Central nervous system
CRP: C reactive protein
CSF: Cerebrospinal fluid
DWI: Diffusion weighted imaging
ESR: Erythrocyte sedimentation rate
ETC: Electron transport chain
FA: Fluorescein angiography
FAF: Fundus autofluorescence
FDG-PET: Fludeoxyglucose positron emission tomography
FLAIR: Fluid-attenuated inversion recovery
GCA: Giant cell arteritis
GCL: Ganglion cell layer
LE: Left eye
LHON: Leber hereditary optic neuropathy
MELAS: Mitochondrial encephalomyopathy, lactic acidosis, and stroke-like episodes syndrome
MOG: Myelin oligodendrocyte glycoprotein
MRI: Magnetic resonance imaging
MS: Multiple sclerosis
mtDNA: Mitochondrial DNA
MT-TL1: Mitochondrially encoded tRNA leucine 1
NAION: Non-arteritic ischemic optic neuropathy
NMO: Neuromyelitis optica
NMOSD: Neuromyelitis optica spectrum disorders
OCT: Ocular coherence tomography
PCR: Polymerase chain reaction
RE: Right eye
RNFL: Retinal nerve fiber layer
SLE: Stroke-like episode
STIR: Short tau inverion recovery
tRNA: Transfer RNA
