# Supplementary figures and images for: Ischemic optic neuropathy as first presentation in patient with m.3243 A > G MELAS classic mutation
Source: BMC Neurol. 2023 Apr 24;23:165. doi: 10.1186/s12883-023-03198-3 (PMC10123965; doi:10.1186/s12883-023-03198-3)

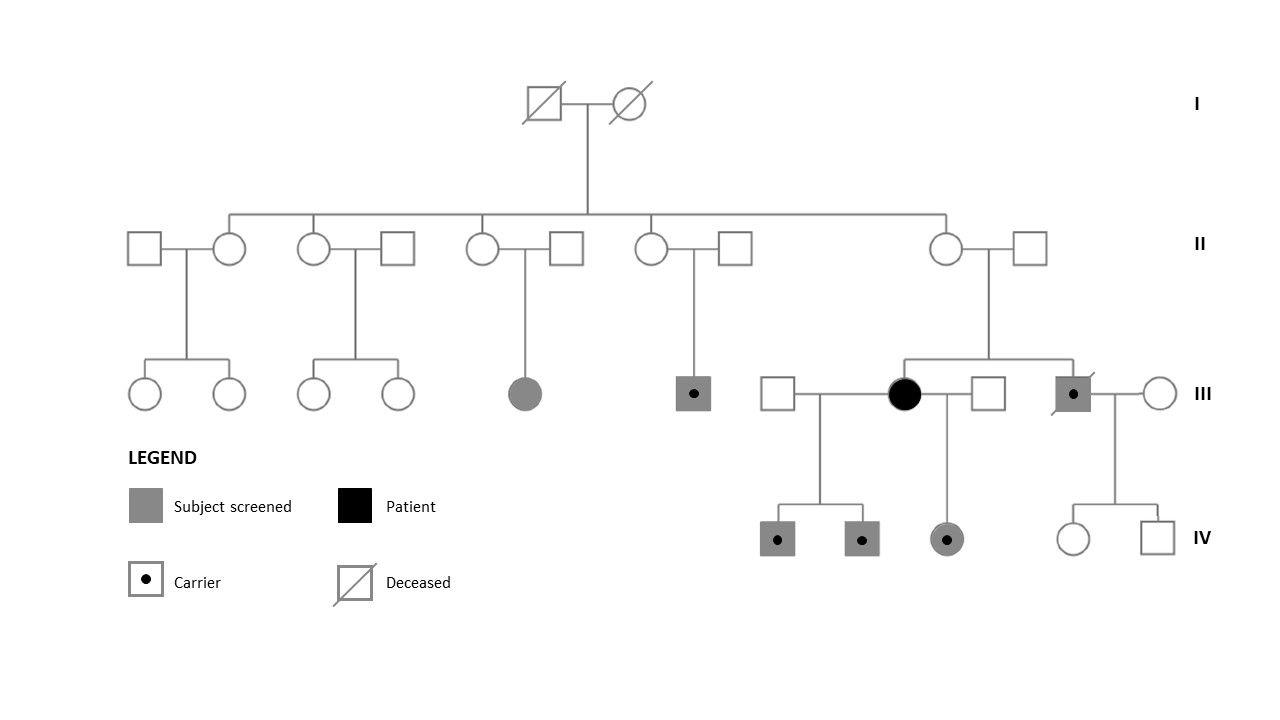

Supplement: Supplementary file 1 — Additional file 1. Supplementary materials. Family history and pedigree. [file 12883_2023_3198_MOESM1_ESM.jpg]
